# Murine Flt3L-derived dendritic cell mediated early immune responses are critical to controlling cell-free HTLV-1 infection

**DOI:** 10.1186/1742-4690-8-S1-A188

**Published:** 2011-06-06

**Authors:** Saifur Rahman, Zafar K Khan, Brian Wigdahl, Stephen R Jennings, Frederic Tangy, Pooja Jain

**Affiliations:** 1 Department of Microbiology and Immunology, and the Drexel Institute for Biotechnology and Virology Research, Drexel University College of Medicine, Doylestown, PA, 18902, USA; 2 Department of Microbiology and Immunology, and the Institute for Molecular Medicine and Infectious Disease, Drexel University College of Medicine, Philadelphia, PA, 19102, USA; 3 Unité de Génomique Virale et Vaccination, URA-3015, Centre National de la Recherche Scientifique (CNRS), Institut Pasteur, Paris, France

## 

Human T-cell leukemia virus type 1 (HTLV-1) is associated with two immunologically distinct diseases: HTLV-1-associated myelopathy/tropical spastic paraparesis and adult T-cell leukemia. We observed previously that depletion of dendritic cells (DCs) in CD11c-DTR transgenic mice followed by infection with cell-free virus led to greater proviral and Tax mRNA loads and diminished the cellular immune response compared to mice infected with cell-associated virus. To understand the significance of these in vivo results and explore the host pathogen interaction between dendritic cells and cell-free HTLV-1, we used Fms-like tyrosine kinase 3 ligand (Flt3L) cultured mouse bone marrow-derived DCs (FL-DCs) and chimeric HTLV-1. Phenotypically, the FL-DCs upregulated expression of surface markers (CD80, CD86, and MHC class II) on infection; however, the level of MHC class I remained unchanged. We performed kinetic studies to understand viral entry, proviral integration, and expression of the viral protein Tax. Multiplex cytokine profiling revealed production of an array of proinflammatory cytokines and type 1 IFN (IFN-α) by FL-DCs treated with virus. Virus-matured FL-DCs stimulated proliferation of autologous CD3+ T cells as shown by intracellular nuclear Ki67 staining and produced IFN-γ when cultured with infected FL-DCs. Gene expression studies using type 1 IFN-specific and DC-specific arrays revealed upregulation of interferon-stimulated genes, most cytokines, and transcription factors but a distinct downregulation of many chemokines. Overall, these results highlight the critical early responses generated by FL-DCs on challenge with cell-free chimeric HTLV-1.

